# Differential effects of glycation on protein aggregation and amyloid formation

**DOI:** 10.3389/fmolb.2014.00009

**Published:** 2014-09-02

**Authors:** Clara Iannuzzi, Gaetano Irace, Ivana Sirangelo

**Affiliations:** Department of Biochemistry, Biophysics and General Pathology, Seconda Università degli Studi di NapoliNaples, Italy

**Keywords:** amyloid aggregation, protein glycation, AGEs, protein misfolding, amyloidosis

## Abstract

Amyloids are a class of insoluble proteinaceous substances generally composed of linear un-branched fibrils that are formed from misfolded proteins. Conformational diseases such as Alzheimer's disease, transmissible spongiform encephalopathies, and familial amyloidosis are associated with the presence of amyloid aggregates in the affected tissues. The majority of the cases are sporadic, suggesting that several factors must contribute to the onset and progression of these disorders. Among them, in the past 10 years, non-enzymatic glycation of proteins has been reported to stimulate protein aggregation and amyloid deposition. In this review, we analyze the most recent advances in this field suggesting that the effects induced by glycation may not be generalized as strongly depending on the protein structure. Indeed, being a post-translational modification, glycation could differentially affects the aggregation process in promoting, accelerating and/or stabilizing on-pathway and off-pathway species.

## Protein aggregation and amyloid formation

Neurodegenerative disorders, including Alzheimer's, Parkinson's, amyotrophic lateral sclerosis and prion diseases are debilitating and so far incurable disorders that demand intensive research. In these diseases, misfolding, aggregation, and precipitation of proteins seem to be directly related to neurotoxicity (Dobson, 2003; Chiti and Dobson, 2006). Specifically, the physiological alterations are associated with the formation of fibrillar aggregates, referred to as amyloid fibrils, that usually accumulate in the extracellular space of tissues or also as intracellular deposits (Stefani and Dobson, 2003; Taylor et al., 2005). Protein molecular assembly is characterized by several events like conformational changes and intermolecular interactions which strongly affect each other. The hierarchy of all these mechanisms and their extent depends on several physical and chemical parameters such as temperature, pH, ionic strength, and addition of denaturants. Until very recently, it was thought that only a small number of polypeptide chains associated with clinical disorders were able to form amyloid fibrils. However, a number of recent studies have shown that proteins unrelated to diseases, under suitable conditions, can form aggregates *in vitro* with structural and cytotoxic properties that closely resemble those of the amyloid fibrils formed in diseased tissues (Litvinovich et al., 1998; Fandrich et al., 2001; Sirangelo et al., 2004, 2009; Iannuzzi et al., 2013a). These observations have led to the idea that the ability to form amyloid fibrils is a generic property of polypeptide chains irrespective of their amino acid sequence and caused by stable interactions involving primarily the common polypeptide backbone. Despite major differences in the sequences and three-dimensional structures of the peptides and proteins involved, the fibrillar forms of the aggregates share a common ultrastructure (Diaz-Avalos et al., 2003; Nelson et al., 2005; Fitzpatrick et al., 2013). They usually consist of a number (typically 2–6) of protofilaments, each about 2–5 nm in diameter, that are often twisted around each other to form super-coiled ropelike structures typically 7–13 nm in width or that laterally associate to form long ribbons that are 2–5 nm thick and up to 30 nm wide (Serpell et al., 2000). X-ray diffraction analysis has indicated that the characteristic structure, i.e., the β-cross motif, is formed by β-strands oriented perpendicular to the long axis of the fibril, and β-sheets propagating in the fibril direction (Sunde and Blake, 1997; Makin and Serpell, 2002; Maji et al., 2009). These findings suggest that a common molecular mechanism could underlie the aggregation process of the different proteins involved in misfolding diseases (Kopito, 2000; Dobson, 2001).

Three major factors have been identified as important parameters in the conversion of a protein into aggregates; these are high hydrophobicity, high propensity to convert from α-helical to β-sheet structure, and low net charge (Konno, 2001; Ciani et al., 2002; Tjernberg et al., 2002; Chiti et al., 2003; Tartaglia et al., 2008). Protein destabilization favors the formation of partially unfolded conformations that are highly prone to aggregation (Uversky and Fink, 2004). In most cases, protein destabilization is facilitated by amino acid mutations which also increase the structural flexibility of the peptide chain; however, other proteins are amyloidogenic even in the wild type form (Hurle et al., 1994; Goedert et al., 2000; Quintas et al., 2001; Niraula et al., 2002; Iannuzzi et al., 2007; Infusini et al., 2012, 2013). It has been suggested that protein folding and protein aggregation, despite being distinct processes, are in competition each other and the environmental conditions dictate which one is favored for a given polypeptide chain (Tartaglia and Vendruscolo, 2010). On this basis, extensive studies have been carried out *in vitro* to investigate the nature of the transition between natively folded states and soluble aggregate-precursor states, and between the latter and mature amyloid fibrils and the factors affecting all of these (Wiseman et al., 2005). Recent data indicate that these dangerous aggregation-prone states, although similar to the native conformation, display altered surface charge distribution, alternative β-sheet topologies and increased solvent exposure of hydrophobic surfaces and of aggregation-prone regions of the sequence (De Simone et al., 2011). The propensity of normally folded proteins to form amyloid-like fibrils increases in conditions that allow the protein to break the major unfolding energy barrier, favoring partial unfolding of the native state. These include low pH, high temperature, or the presence of organic solvents (Guijarro et al., 1998; Villegas et al., 2000). However, increasing evidence is now accumulating that folded proteins also retain a significant tendency to aggregate with no need for unfolding as first obligatory step (Plakoutsi et al., 2004; Bemporad and Chiti, 2009).

**Figure 1:**
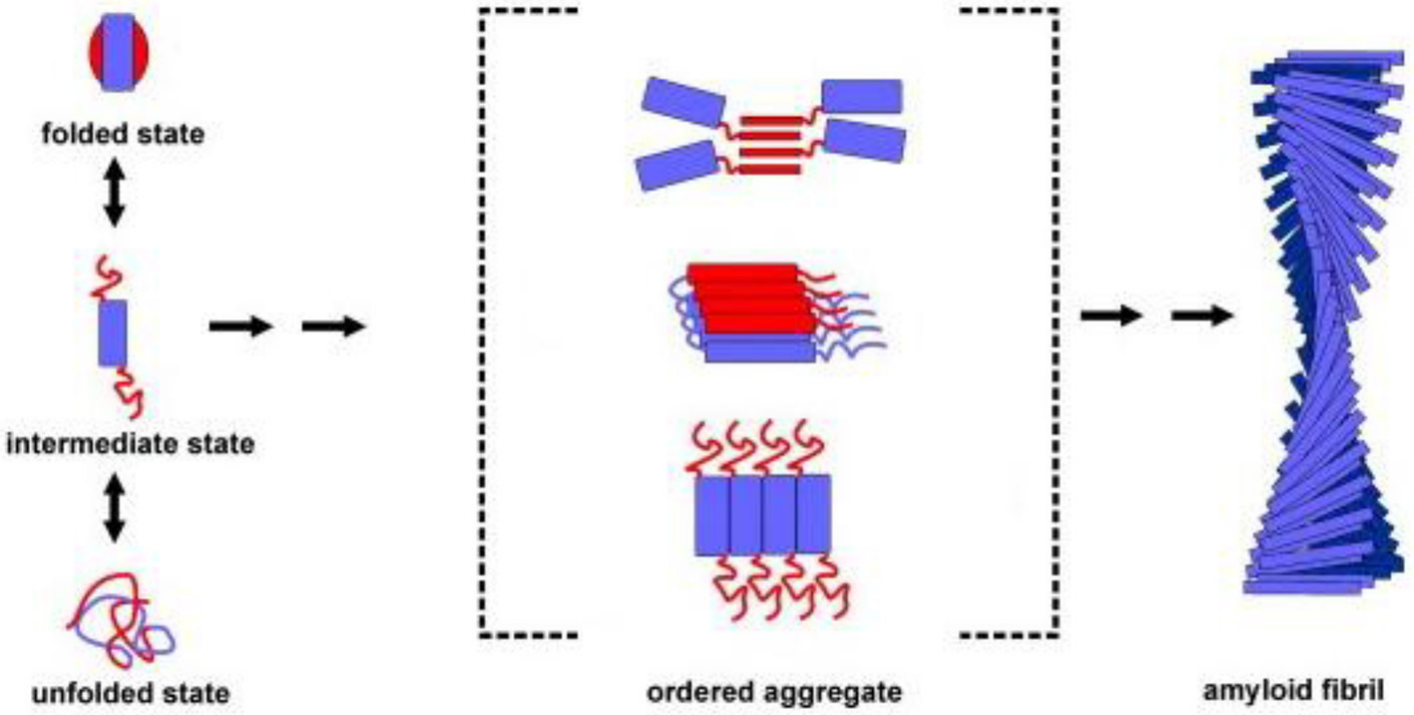
**Association of two or more non-native peptide/protein molecules forming highly ordered, fibrillar aggregates**.

**Figure 2:**
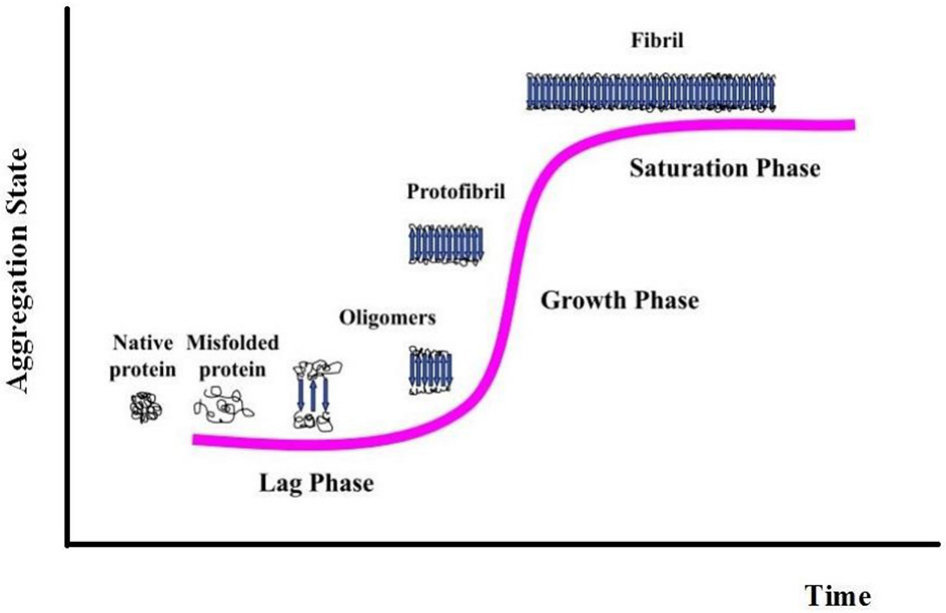
**Nucleation-dependent fibril formation process**.

Protein aggregation begins with the appearance of aggregation nuclei, whose growth is considered the rate-limiting step of the process, which has many characteristics of a nucleation-dependent polymerization mechanism (Kelly, 1998) (Figures 1, 2). These species, generally indicated as protofibrils or soluble oligomeric intermediates, appear as globules of 2.5–5.0 nm in diameter or larger, with an intrinsic tendency to further assemble into pore-like annular and tubular structures (Lashuel et al., 2002; Poirier et al., 2002). Once a nucleus is formed, fibril growth is thought to proceed rapidly by further association of either monomers or oligomers with the nucleus (Cohen et al., 2012).

While insoluble aggregates correlate with disease progression, there are increasing evidences that the initiating and most toxic events are caused by prefibrillar forms rather than mature fibrils. These results have led to the idea that molecular basis of cell and tissue impairment may be related to the transient appearance of prefibrillar assemblies, under conditions where their intracellular levels increase as a consequence of dysfunctions in cellular clearance machineries (Stefani, 2012). The specific mechanism by which these species appear to mediate their toxic effects is not completely understood; probably toxicity is mediated by common structural features shared by prefibrillar precursors (Kayed et al., 2003; Bucciantini et al., 2004; Malmo et al., 2006; Cecchi and Stefani, 2013).

## Protein glycation and amyloidosis

Although the aggregation process of amyloidogenic proteins has been widely studied *in vitro* and many physiological (environmental and genetic) factors involved have been identified, the molecular mechanisms underlying the formation of aggregates *in vivo* and in pathological conditions are still poorly understood. The majority of neurodegenerative diseases are sporadic, suggesting that other factors must contribute to the onset and progression of these disorders. Post-translational modifications are known to affect protein structure and function. Some of these modifications might affect proteins in detrimental ways and lead to their misfolding and accumulation. Reducing sugars play an important role in modifying proteins, forming advanced glycation end-products (AGEs) in a non-enzymatic process named glycation. This process is different from glycosylation; indeed these two post-translational modifications affect the structure of the target protein in a different way. Glycosylation is a selective protein modification, driven by specific enzymes, that is generally associated to a gain of function (or stabilization) of the target protein. Non-enzymatic glycation is a non-selective modification and it is generally associated to a loss of function of the target protein due to modifications of its native structure. While glycosylation is a well controlled cellular mechanism, non-enzymatic glycation only depends on the exposure of free amino-groups in the polypeptide chain, concentration of the sugar and oxidative conditions.

**Figure 3:**
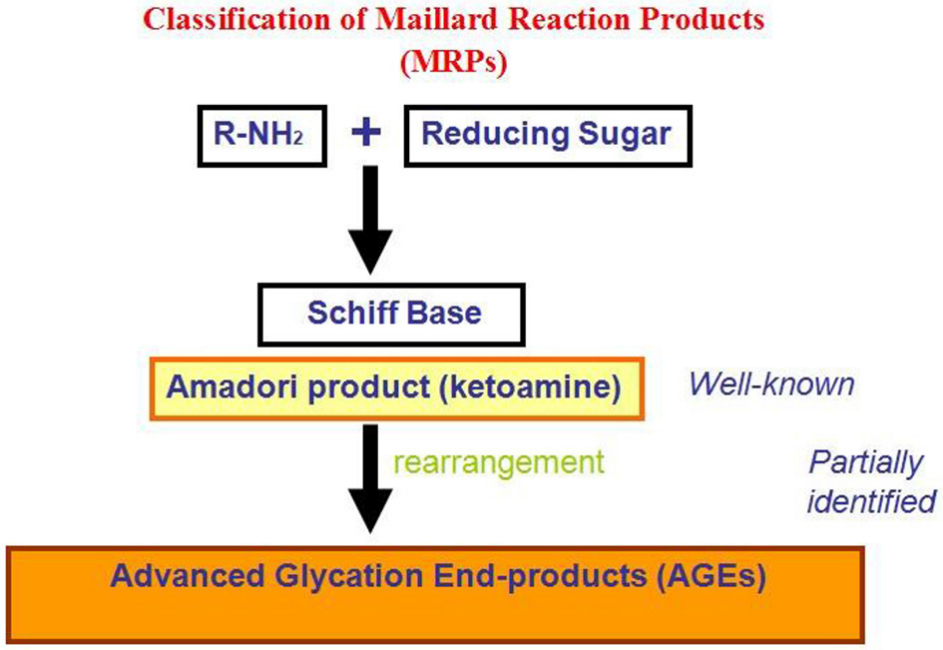
**Classification of non-enzymatic glycation reaction products**.

Recently, much attention has been devoted to the role played by non-enzymatic glycation of proteins in stimulating amyloid aggregation and toxicity. Proteins in amyloid deposits are found often glycated suggesting a direct correlation between protein glycation and amyloidosis (Miyata et al., 1993; Kikuchi et al., 2000; Munch et al., 2000; Dukic-Stefanovic et al., 2001; Shults, 2006). Glycation reactions are common to all cell types: glycated products slowly accumulate *in vivo* leading, besides cellular modifications involved in the aging process, to several different protein dysfunctions (Lyons et al., 1991; Miyata et al., 1999; Gul et al., 2009). The process begins with a nucleophilic addition reaction between a free amino group of a protein and a carbonyl group of a reducing sugar, forming a reversible intermediate product (Schiff's base). Side-chains of arginine and lysine residues, the N-terminus amino group of proteins, and thiol groups of cysteine residues, are the main targets of protein glycation. The process depends on several conditions, such as the concentration and reactivity of the glycation agent, the presence of catalytic factors (metals, buffer ions and oxygen), the physiological pH, temperature and the half-life of each protein. All reducing sugars can participate in glycation reactions and, between them, D-ribose is the most active and its intracellular level can be quite high. D-glucose is the least reactive and its intracellular concentration is negligible, while dicarbonyl compounds are far more reactive. The levels of D-ribose in the blood are estimated around 20 mg/L in healthy individuals while D-glucose 6–10 g/L. Once formed, the Schiff's base can turn into a stable ketoamine by Amadori rearrangement (Figure 3). This reaction is reversible depending on the concentration of the reactants. The late-stage of the process is an irreversible cascade of reactions involving dehydration, hydrolysis, and other rearrangements leading to the formation of AGEs. AGEs products are considered to be a marker of several diseases, such as arteriosclerosis, renal failure, Alzheimer disease, or diabetes, although they normally increase in aging (Vlassara, 2005).

**Figure 4:**
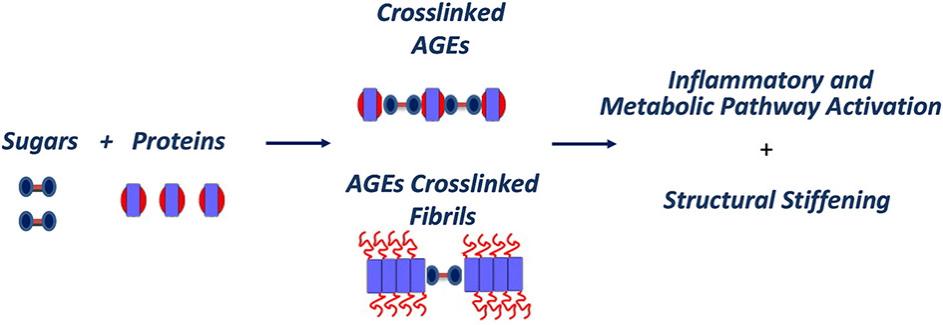
**AGEs pathway in aging and amyloid diseases**.

Indeed, protein glycation has been considered an age related problem influencing mainly extracellular proteins, such as collagen and elastin, which are located outside the cells and provide strength and flexibility to the tissues. AGEs formation can interfere not only with the regular functioning of the proteins to which they are attached but also induce the formation of covalent cross-links with close proteins. This process is gradual, so that cross-links accumulate over the years on the longest-lived extracellular proteins, which do not get cleared very often; clear evidence of this is found in the extracellular collagen and elastin (Furber, 2010). The observation that proteins in amyloid deposits, such as β-amyloid, tau, prions and transthyretin, are often found glycated in patients suggests a direct correlation between protein glycation and amyloid formation. This is thought to be associated with an increased protein stability through the formation of cross-links that stabilize protein aggregates (Figure 4). Also, glycation affects the structure and the biological activity of proteins as well as their degradation process (Shaklai et al., 1984; Mendez et al., 2005) and, being an abnormal modification, it has been found to induce some proteins to misfold and, thus, promote protein aggregation (Vitek et al., 1994; Chellan and Nagaraj, 1999; Verzijl et al., 2002; Bouma et al., 2003).

Moreover, once proteins become glycated at their exposed lysine residues, clearance by the ubiquitin-proteasome system would be impaired because ubiquitination of lysine residues, a modification that targets proteins to the proteasome for degradation, might be impeded. Thus, accumulation of proteins as aggregates or as depositions or inclusions in tissues might be favored after glycation.

However, in addition to directly affecting protein structure and function, AGEs also exert cellular effects mediated by specific AGEs receptors (RAGE), as well as macrophage scavenger receptors, MSR type II, OST-48, 80K-H, galectin-3, and CD36 (Vlassara et al., 1995; Li et al., 1996; Ohgami et al., 2002; Stern et al., 2002). Indeed, glycation may be responsible, via RAGE, for an increase in oxidative stress and inflammation through the formation of reactive oxygen species and the activation of the nuclear transcription factor-κ B generally associated to amyloid toxicity (Xie et al., 2013).

## Differential effects of glycation on protein aggregation

Several proteins related and not related to misfolding diseases have been so far examined to investigate the effect of glycation on their propensity to aggregate and form amyloid structure.

### Aβ-peptide

Vitek et al. (1994) observed, for the first time, that plaque fractions of AD brains contained about three-fold more AGE adducts than preparations from healthy, age-matched controls. They showed that the *in vivo* half-life of β-amyloid is prolonged in AD, resulting in greater accumulation of AGE modifications which may, in turn, act to promote accumulation of additional amyloid. Moreover, AGE-modified Aβ peptide-nucleation seeds accelerated aggregation of soluble Aβ peptide compared to non-modified seed material (Vitek et al., 1994). Successively, Munch et al. (1997, 2000) reported that glycation promotes *in vitro* amyloid aggregation of Aβ peptide, probably because of crosslinking through AGEs formation. Further studies revealed that glycation is not only capable of enhancing the rate of formation of amyloid, oligomers and protofibrils but also of increasing the size of the aggregates (Chen et al., 2006). The fibrillar aggregates formed upon glycation were not cytotoxic, thus glycation in the Aβ peptide seems to strongly reduce its toxicity (Fernandez-Busquets et al., 2010).

### β2-microglobulin

Also in the case of β2-microglobulin, glycation seems to promote amyloid aggregation. In particular, D-ribose interacts with human β2-microglobulin to generate AGEs that form aggregates in a time-dependent manner. Ribosylated β2-microglobulin molecules are highly oligomerized compared with the unglycated protein, and have granular morphology. Such ribosylated β2-microglobulin aggregates show significant cytotoxicity to both human SH-SY5Y neuroblastoma and human foreskin fibroblast FS2 cells and induce the formation of intracellular reactive oxygen species (Kong et al., 2011). By contrast, modification of β2-microglobulin with D-glucose was reported to inhibit fibril extension *in vitro* (Hashimoto et al., 1999).

### Insulin

A different effect has been observed for glycated insulin. This protein is intimately associated with glycaemia and is vulnerable to glycation by glucose and other highly reactive carbonyls especially in diabetic conditions. (Brange et al., 1997). *In vitro* experiments have shown that glucose is able to produce glycated bovine insulin on Lys29 in the C-terminal region of chain B and on N-terminus of chains A and B. Glucose produces glycated bovine insulin adducts with different structural features depending on the experimental conditions. In particular, in reducing conditions glycation produces higher levels of insulin oligomerization and, therefore, accelerates amyloid formation. On the contrary, in non-reducing conditions, glycation inhibits amyloid formation in a way proportional to the glycation extent (Alavi et al., 2013). Probably, under these conditions, insulin adducts possess a higher internal dynamics that prevent formation of the rigid cross-β core structure thus reducing the ability to form fibrils. Methylglyoxal is able to produce glycated human insulin in a single site, i.e., Arg46 of the B-chain. This modification induces the formation of native-like aggregates and reduces the ability to form fibrils by blocking the formation of the seeding nuclei. These aggregates are small, soluble, non-fibrillar and retain a native-like structure. The lag phase of the nucleation-dependent polymerization process increased as a function of methylglyoxal concentration. In this case glycation preserved insulin native conformation, blocking the α-helix to β-sheet transition thus leading to a reduced fibril formation. Again, the effects may be ascribed to a higher dynamics in glycated insulin leading to impairment in the formation of the rigid cross-β core structure. Taken together, these results showed that methylglyoxal-induced glycation reduces insulin fibril formation and promotes the population of oligomeric states (Oliveira et al., 2011).

### Cytochrome c

Cytochrome c (Cyt c) was also used as a model protein to study the impact of glycation on protein structure, stability, and ability to form aggregates. Methylglyoxal has been shown to covalently modify Cyt c at a single arginine residue and induces early conformational changes that lead to the formation of native-like aggregates without promoting amyloid formation. Oligomerization occurs due to localized protein structural changes, which induce a decrease in the conformational stability of the modified protein. Consequently, the aggregation process starts directly by monomer addition in a way that is thermodynamically and kinetically favored. Furthermore, partially unfolded species are formed, but they do not seem to be implicated in the aggregation process. Interestingly, the glycated Cyt c unfolded species are an off pathway by-product and, for this reason, they do not promote the amyloidogenic aggregation pathway (Oliveira et al., 2013).

### α-synuclein

Glycation of α-synuclein is a factor involved in the aggregation of the protein into Parkinson's disease and in the formation of Lewy bodies (LB). Glycation was first reported to be present in substantia nigra and locus coeruleus of peripheral LB (Vicente and Outeiro, 2010). The protein has 15 lysine residues making it a target for glycation at multiple sites (Padmaraju et al., 2011). Lee and collaborators found that methylglyoxal induces oligomerization of α-synuclein and inhibits the formation of amyloid fibrils. Moreover, protein fibrillization was also significantly suppressed by the seeding of modified α-synuclein species (Lee et al., 2009). Similar results were obtained with D-ribose: ribosylation of α-synuclein promotes the formation of molten globule-like aggregates which caused cells oxidative stress and resulted in high cytotoxicity (Chen et al., 2010).

### Lysozyme

Also hen egg white lysozyme (HEWL) has been used to study the impact of glycation on protein structure and aggregation. HEWL is a structural homolog of human lysozyme, responsible for systemic amyloidosis disease and, for this reason, considered a very good model. HEWL undergoes glycation *in vitro* and potential glycation sites are considered to be the N-terminal α-amino group, ε-amino group of lysine residues and guanidino group of arginine residues (Tagami et al., 2000).

Glycation of HEWL has been tested over a prolonged period in the presence of D-glucose, D-fructose and D-ribose (Fazili and Naeem, 2013; Ghosh et al., 2013). Glycation has been found to promote the formation of cross-linked oligomers in HEWL instead of amyloid aggregates and, among the tested sugars, D-ribose resulted the most effective one. Glycation in HEWL has been shown to promote at first an alpha to β transition and then, prolonged glycation induced the formation of cross-linked β-sheet rich oligomers which are amorphous and globular in nature.

### Albumin

Also human and bovine serum albumin (BSA) have been shown to be efficiently glycated *in vitro* by D-ribose and, in this case, glycation has been shown to promote amyloid aggregation (Bouma et al., 2003; Sattarahmady et al., 2007). Although BSA is a highly soluble protein rich in helical structure, glycation promotes strong conformational changes affecting both secondary and tertiary structure. Indeed, it has been observed a strong reduction of the helical content and, subsequently, the formation of β-rich aggregates that rapidly evolve to the formation of amyloid fibrils.

Amyloid-like aggregates of glycated BSA are able to induce high cytotoxicity that trigger cell death by activation of cellular signaling cascades. Indeed, independent experiments have shown that aggregates of glycated BSA are able to induce oxidative stress ROS mediated and apoptosis in both neurotypic SH-SY5Y and MCF-7 cells (Wei et al., 2009; Khan et al., 2013).

### W7FW14F apomyoglobin

Recently, it has been shown that glycation of the amyloidogenic apomyoglobin mutant W7FW14F significantly accelerates the amyloid fibrils formation providing evidence that glycation actively participates to the process affecting the reaction kinetics (Iannuzzi et al., 2013b). Moreover, it has been examined the effect of glycation on wild type apomyoglobin and preliminary results indicate that, for this protein, AGEs formation does not trigger amyloid aggregation, thus suggesting that the presence of amyloidogenic sequences in a misfolded protein is crucial for predisposing the protein to amyloid aggregation (unpublished data). These data indicate that a synergy between predisposing factor, i.e., aggregation propensity, and AGEs induced cross-links formation may be a strongly relevant factor in addressing the formation of amyloid structure.

The differences observed in the protein models so far studied might be a consequence of the inherent properties of the native structure of each protein or structural changes induced by AGE modifications as result of different glycation agents. In most of the cases mentioned above, fibrillation enhancement is achieved by modifying amyloidogenic proteins with glycating sugars like glucose or fructose while small and highly reactive carbonyls like methylglyoxal are apparently more prone to reduce fibril formation. This suggests that different glycation agents lead to specific structural constraints that have a major role in protein fibrillation kinetics. Moreover, some glycated proteins undergo oligomerization without promoting amyloid fibril formation and this can be related to the aggregation behavior of some amyloidogenic proteins upon glycation. In fact both insulin and α-synuclein, which are involved in amyloid diseases, show decreased amyloid fibril formation after glycation and both significantly retain the native three dimensional structure during the aggregation process. Overall, glycation of amyloidogenic proteins can lead to a shift from an amyloidogenic pathway to a native-like aggregation through a process that is thermodynamically and kinetically favored.

## Conclusions and perspectives

The above referred considerations make the study of AGEs one of the most important areas of biomedical research today. Several questions remain to be answered: whether glycation of susceptible proteins is a triggering event or just a result of its reactivity toward low-turnover aggregated species, which are highly insoluble and protease-resistant, remains controversial. Several studies suggest that glycation may be an early event promoting or accelerating abnormal protein deposition, followed by increased protease resistance and insolubility. Regardless of the chronology of AGEs formation, it is known that its accumulation is related to sustained inflammatory responses and oxidative stress, which is a common feature in many neurodegenerative disorders. Glycation may then be understood as a dynamic contributor to these multifactorial diseases by promoting, accelerating or stabilizing pathological protein aggregation and inducing responses leading to cell dysfunction, damage and death. Thus, it will be important to further investigate the biochemical effects induced by the interaction of AGEs-modified proteins with cells, such as, the activation of oxidative stress signaling pathway and inflammatory response.

### Conflict of interest statement

The Associate Editor, Dr. Piero Andrea Temussi, declares that despite having collaborated with author Clara Iannuzzi in the past 2 years, there has been no conflict of interest during the review and handling of this manuscript. The authors declare that the research was conducted in the absence of any commercial or financial relationships that could be construed as a potential conflict of interest.
